# An exploration of the subjective social status construct in patients with acute coronary syndrome

**DOI:** 10.1186/s12872-018-0759-7

**Published:** 2018-02-06

**Authors:** Karen L. Tang, Louise Pilote, Hassan Behlouli, Jenny Godley, William A. Ghali, for the GENESIS-PRAXY investigators

**Affiliations:** 1 Department of Medicine, University of Calgary, 3330 Hospital Drive NW, Calgary, AB T2N 4N1 Canada; 2 Division of Clinical Epidemiology, Research Institute of McGill University Health Centre, 687 Pine Ave West, Montreal, Quebec, H3A 1A1 Canada; 3 Division of General Internal Medicine, McGill University Health Centre, 687 Pine Ave West, Montreal, Quebec, H3A 1A1 Canada; 4 Department of Sociology, University of Calgary, 2500 University Drive NW, Calgary, AB T2N 1N4 Canada; 5 O’ Brien Institute for Public Health, University of Calgary, 3280 Hospital Drive NW, Calgary, AB T2N 4Z6 Canada; 6 Department of Community Health Sciences, University of Calgary, 3280 Hospital Drive NW, Calgary, AB T2N 4Z6 Canada

**Keywords:** Social class, Socioeconomic status, Cardiovascular disease, Readmissions

## Abstract

**Background:**

Perception of low subjective social status (SSS) relative to others in society or in the community has been associated with increased risk of cardiovascular disease. Our objectives were to determine whether low SSS in society was associated with barriers to access to care or hospital readmission in patients with established cardiovascular disease, and whether perceptions of discordantly high SSS in the community modified this association.

**Methods:**

We conducted a prospective cohort study from 2009 to 2013 in Canada, United States, and Switzerland in patients admitted to hospital with acute coronary syndrome (ACS). Data on access to care and SSS variables were obtained at baseline. Readmission data were obtained 12 months post-discharge. We conducted multivariable logistic regression to model the odds of access to care and readmission outcomes in those with low versus high societal SSS.

**Results:**

One thousand ninety patients admitted with ACS provided both societal and community SSS rankings. The low societal SSS cohort had greater odds of reporting that their health was affected by lack of health care access (OR 1.48, 95% CI 1.11, 1.97) and of experiencing cardiac readmissions (1.88, 95% CI 1.15, 3.06). Within the low societal SSS cohort, there was a trend toward fewer access to care barriers for those with discordantly high community SSS though findings varied based on the outcome variable. There were no statistically significant differences in readmissions based on community SSS rankings.

**Conclusion:**

Low societal SSS is associated with increased barriers to access to care and cardiac readmissions. Though attenuated, these trends remained even when adjusting for clinical and sociodemographic factors, suggesting that perceived low societal SSS has health effects above and beyond objective socioeconomic factors. Furthermore, high community SSS may potentially mitigate the risk of experiencing barriers to access to health care in those with low societal SSS, though these associations were not statistically significant. Subjective social status relative to society versus relative to the community seem to represent distinct concepts. Insight into the differences between these two SSS constructs is imperative in the understanding of cardiovascular health and future development of public health policies.

**Electronic supplementary material:**

The online version of this article (10.1186/s12872-018-0759-7) contains supplementary material, which is available to authorized users.

## Background

Individuals with lower objective socioeconomic status (SES), such as those with lower income, lower educational attainment, or working in lower status occupations, consistently experience increased mortality, increased prevalence of coronary artery disease, and worse prognosis after an acute coronary syndrome (ACS) event relative to those with higher objective SES [1–6]. Because these disparities exist not just when comparing the rich versus the poor but along a gradient even among those with relatively high status [7], absolute material deprivation does not fully explain the disparities in outcomes. Perceptions of relative differences in social standing may therefore contribute to health in important ways.

An individual’s perceived position on the social hierarchy has been termed “subjective social status” (SSS) [8]. In health research, SSS is generally measured on a vertical 10-rung ladder representing either the society or the specific country in which the tool is being used (the “societal ladder”), or the community as defined by the participant (the “community ladder”) [9]. Though the two ladders are correlated, sharing 50% of variance [10], preliminary evidence suggests that they may be distinct [10], with individuals choosing their rankings on the societal ladder primarily based on wealth, occupation and education, whereas less objective characteristics such as altruism seem to take priority in choosing one’s position on the community ladder [9]. Most studies on SSS and cardiovascular health consider the societal ladder only [11–16]. Only one cross-sectional study considers the combined effect of both ladder rankings on the prevalence of cardiovascular risk factors [17].

Though there are large numbers of studies on the association between SSS and mental health, self-rated health, risk-taking behaviours, and cardiovascular risk factors [18], there are few studies on the association of SSS and outcomes and prognosis in those with established cardiovascular disease [19, 20]. We undertook a study in patients hospitalized with ACS to address whether low societal SSS is associated with increased barriers to access to health care and increased readmissions within 1 year after discharge from hospital. In addition, we sought to examine whether community SSS modified the association between societal SSS and access to care and readmissions in this cohort of patients. Because high community SSS may reflect increased social support, social capital, and altruism, each of which tends to confer cardioprotective benefit [21–23], we hypothesized that having high community SSS might mitigate the risk of barriers to access to care and readmissions especially in those with low societal SSS. Our findings shed light on the construct of SSS and the differences between societal versus community SSS, and they also raise interesting mechanistic questions regarding the relationships between social determinants of health and outcomes.

## Methods

### Study population

Study participants were from the GENESIS PRAXY (Gender and Sex Determinants of Cardiovascular Disease: From Bench to Beyond Premature Acute Coronary Syndrome) prospective cohort study of patients hospitalized with ACS. Patients were enrolled into the study from January 2009 to April 2013, from 24 participating hospitals across Canada, 1 hospital in the United States, and 1 in Switzerland. Inclusion criteria were: 1) Adults aged 18 to 55 years; 2) Fluency in English or French; 3) Ability to provide informed consent; and 4) Diagnosis of ACS by the treating physician, meeting at least one of the following two criteria: (a) ECG changes in two or more contiguous leads (transient ST segment elevations of ≥1 mm, ST segment depressions of ≥1 mm, new T wave inversions of ≥1 mm, pseudo-normalization of previously inverted T waves, new Q-waves [1/3 the height of the R wave or ≥0.04 s], new R > S wave in lead V1, or new left bundle branch block); (b) Increase in cardiac enzymes (CK-MB or CPK (if CK-MB not available) > 2× the upper limit of the hospital’s normal, positive troponin I, or positive troponin T) [24]. Each study participant provided written informed consent.

### Data collection

Data were collected using questionnaires and full chart review at baseline and at 12 months. The questionnaire was self-administered at baseline and administered by a research nurse over the telephone at 12 months. Details regarding study methods have been previously published [25]. Patients were asked to complete both the community and societal MacArthur Scales of Subjective Social Status on the baseline questionnaire [9]. Sociodemographic information including age, sex, employment, household income, and social supports were also obtained from this baseline questionnaire. Clinical factors including type of ACS experienced, clinical comorbidities, and in-hospital complications were obtained from baseline chart review.

Outcome measures included access to care and readmissions to hospital. Access to care variables, such as whether patients have a regular family doctor and whether (and what types of) difficulties were experienced in accessing care, were obtained from the baseline questionnaire. Readmission information were obtained via telephone follow-up and chart review 12 months after the index hospitalization.

### Statistical analysis

Because the societal SSS ladder has better reliability [26] and is also much more widely studied in the literature compared to the community ladder, the main division of our cohort was based on societal SSS rankings. Three sets of comparisons were made for each analysis: 1) Low societal SSS (ranking lower than median) versus high societal SSS (ranking at least as high as the median); 2) Within the low societal SSS cohort, concordantly low community SSS versus discordantly high community SSS; and 3) Within the high societal SSS cohort, concordantly high community SSS versus discordantly low community SSS.

We compared baseline demographics, clinical characteristics and comorbidities, proportions reporting barriers to access to care, and proportions being readmitted to hospital between the two groups for each of the three sets of comparisons, using Fisher’s exact and Chi-square tests (for proportions) and t-tests (for continuous variables). Both unadjusted and adjusted logistic regression were undertaken, modeling the odds of readmission and four access to care outcomes (no family doctor, difficulty accessing a cardiologist, difficulty accessing routine care, and health affected by lack of access). Adjustment for confounding was undertaken for age, sex, comorbidity count, type of ACS, household income, and employment status. All statistical analyses were performed using SAS Version 9.4 (SAS Institute, Cary, NC).

## Results

### Concordance versus discordance of societal versus community SSS

Of 1213 enrolled patients with ACS, 1090 patients provided both societal and community SSS rankings. The median and mean rankings were 6 (interquartile range, IQR, 4, 7) and 5.5 (standard deviation, SD, 2.1) respectively for the societal ladder and 6 (IQR 5, 7) and 6.0 (SD 2.0) respectively for the community ladder.

**Fig. 1:**
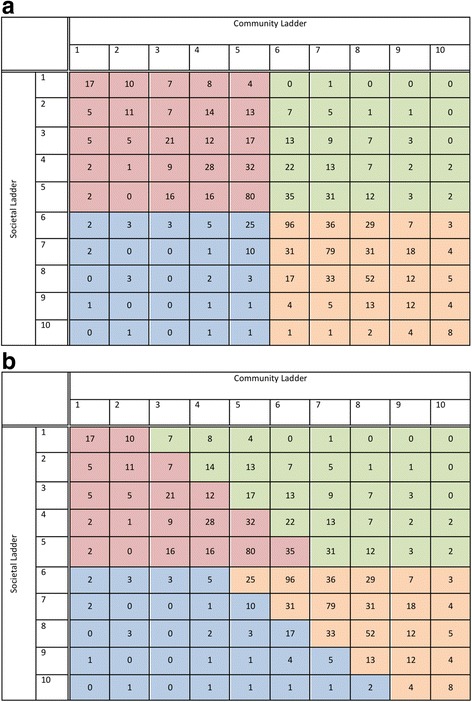
Cross-tabulation of MacArthur Scale of Subjective Social Status societal and community ladders, using the quadrant (**Panel a**) and agreement band (**Panel b**) approaches to divide the sample into four groups. Where Red = Low societal subjective social status (SSS) with concordantly low community SSS; Orange = High societal SSS with concordantly high community SSS; Green = Low societal SSS with discordantly high community SSS; Blue = High societal SSS with discordantly low community SSS

We considered two different approaches to create cohorts based on concordance and discordance in societal and community SSS rankings. The “quadrant approach” (Fig. 1 Panel a) divides the cross-tabulation of societal and community SSS rankings into four quadrants, based on whether rankings were below versus at least as high as the median. A concern with this division is that rankings on a community ladder may need to be interpreted relative to rankings on the societal ladder. For example, an individual with a self-rank of “5” on the societal ladder and “1” on the community ladder would be considered to have concordantly low rankings, when there is considerable difference between these two rankings, while another individual with a self-rank of “5” and “6” respectively would be considered to have discordant rankings despite the difference of only one.

Therefore, we devised and used the “agreement band approach” (Fig. 1 Panel b). Within the low societal group, patients ranking themselves higher on the community ladder by two rungs or more were considered to have discordantly high community rankings; all others in the low societal group were considered to have concordantly low rankings. Similarly, within the high societal group, patients ranking themselves lower on the community ladder by 2 rungs or more were considered to have discordantly low community rankings; all others in the high societal group were considered to have concordantly high rankings. This approach allows consideration of relative rankings on the two ladders. Within the low societal SSS cohort (*n* = 518), 314 patients had concordantly low and 204 had discordantly high community rankings; within the high societal SSS cohort (*n* = 572), 502 had concordantly high and 72 had discordantly low community rankings.

### Low societal SSS versus high societal SSS

Baseline demographics for the low versus high societal SSS cohorts are outlined in Table 1. The median ages (IQR) in the low and high societal SSS cohorts were 49 (45, 53) years and 50 (45, 53) years respectively. Compared with the high societal SSS cohort, a greater proportion of the low societal SSS cohort were females (37.1% vs. 26.9%, *p* < 0.01), smokers (48.5% vs. 32.7%, p < 0.01), had first languages that were neither English nor French (16.9% vs. 11.4%, *p* = 0.04), lived alone (20.5% vs. 11.5%, p < 0.01), had a household income of <$50,000 (50.8% vs. 20.3%, *p* < 0.01), and had a lower proportion who completed post-secondary education (23.0% vs. 38.3%, p < 0.01). The mean social support score on the ENRICHD Social Support Instrument (ESSI) was also lower in those with low societal SSS compared with high societal SSS. The baseline clinical characteristics for the low and the high societal SSS cohorts were similar (Table 2), though a greater proportion of low societal SSS patients had non-ST segment elevation myocardial infarctions, and the comorbidities of diabetes (19.1% vs. 12.4%, *p* < 0.01) and depression (12.8% vs. 8.1%, *p* = 0.01).

**Table 1:** Baseline demographics

		Low Societal SSS *N* = 518, n (%)	High Societal SSS *N* = 572, n (%)	*P*-value
Age (years)	Mean (SD)	48.1 (5.9)	48.3 (5.8)	0.57
Female		192 (37.1)	154 (26.9)	*< 0.01*
Ethnicity	Caucasian	436 (85.8)	496 (88.9)	0.23
	Aboriginal	18 (3.5)	13 (2.3)	
	Chinese	48 (9.5)	39 (7.0)	
	Other	6 (1.2)	10 (1.8)	
First Language	English	288 (58.0)	335 (60.8)	*0.04*
	French	125 (25.2)	153 (27.8)	
	Other	84 (16.9)	63 (11.4)	
Current Smoker		251 (48.5)	187 (32.7)	*< 0.01*
Low Household Income	<$50,000	221 (50.8)	99 (20.3)	*< 0.01*
Education	No degree, certificate, diploma	79 (15.5)	51 (9.0)	*< 0.01*
	High School Diploma	143 (28.1)	128 (22.6)	
	Some Post-Secondary	105 (20.6)	102 (18.0)	
	Completed Post-Secondary	117 (23.0)	217 (38.3)	
	Trades Certificate	65 (12.8)	69 (12.2)	
Employment Status	Currently working	371 (71.6)	500 (87.4)	*< 0.01*
	Student	9 (1.7)	8 (1.4)	0.81
	Homemaker	31 (6.0)	18 (3.2)	*0.03*
	Unemployed	46 (8.9)	17 (3.0)	*< 0.01*
	Leave of Absence	66 (12.7)	32 (5.6)	*< 0.01*
	Disabled	7 (1.4)	3 (0.5)	0.21
	Retired	8 (1.5)	11 (1.9)	0.65
Live alone		106 (20.5)	66 (11.5)	*< 0.01*
Social Support ESSI Sum Score	Mean (SD)	27.0 (7.3)	29.8 (5.9)	*< 0.01*

Abbreviations: *SSS* subjective social status, *SD* standard deviation, *ESSI* ENRICHD Social Support Inventory

Italics: Statistically significant difference, *p*< 0.05

**Table 2:** Baseline clinical characteristics

		Low Societal SSS N = 518, n (%)	High Societal SSS N = 572, n (%)	*P*-value
BMI (kg/m^2^)	Mean (SD)	29.9 (7.2)	29.2 (5.7)	0.08
Type of MI on admission	STEMI	291 (56.2)	344 (60.1)	0.20
	NSTEMI	189 (36.5)	173 (30.2)	*0.03*
	Unstable Angina	28 (5.4)	50 (8.7)	*0.04*
Reperfusion		395 (77.6)	444 (78.6)	0.71
Method of Reperfusion	Primary PCI	182 (44.5)	225 (48.3)	0.28
	Non Primary PCI	205 (50.3)	216 (46.4)	0.28
	Thrombolytics	69 (16.9)	71 (15.2)	0.52
Peak Troponin T, Mean (SD)		8.6 (28.2)	6.1 (18.3)	0.32
Comorbidities	Angina	174 (33.6)	176 (30.8)	0.33
	Cancer	5 (1.0)	17 (3.0)	*0.03*
	Diabetes	99 (19.1)	71 (12.4)	*< 0.01*
	Congestive heart failure	10 (1.9)	10 (1.8)	0.83
	Hypertension	207 (40.0)	208 (36.4)	0.24
	Hyperthyroid	7 (1.4)	10 (1.8)	0.56
	Dyslipidemia	224 (43.2)	228 (39.9)	0.27
	Peripheral Artery Disease	13 (2.5)	5 (0.9)	0.05
	Depression	62 (12.8)	44 (8.1)	*0.01*
	Renal Disease	7 (1.4)	10 (1.8)	0.64
	Previous MI	75 (14.5)	60 (10.5)	0.05
LV Function (%)	Mean (SD)	51.1 (11.5)	51.1 (9.8)	0.99
Complications in hospital	Atrial fibrillation	12 (2.3)	8 (1.4)	0.27
	Angina	25 (4.8)	27 (4.7)	1.00
	Bradycardia	12 (2.3)	10 (1.8)	0.53
	Cardiogenic Shock	3 (0.6)	5 (0.9)	0.73
	Hypotension	14 (2.7)	17 (3.0)	0.86
	Ventricular tachycardia	25 (4.8)	17 (3.0)	0.12
	Ventricular fibrillation	15 (2.9)	20 (3.5)	0.61
	Congestive heart failure	6 (1.2)	12 (2.1)	0.25
	Pericarditis	4 (0.8)	4 (0.7)	1.00
	Reinfarction	4 (0.8)	2 (0.4)	0.43

Abbreviations: *SSS* subjective social status, *SD* standard deviation, *BMI* body mass index, *PCI* percutaneous coronary intervention, *MI*myocardial infarction, *LV* left ventricular

Italics: Statistically significant difference, *p*< 0.05

**Fig. 2:**
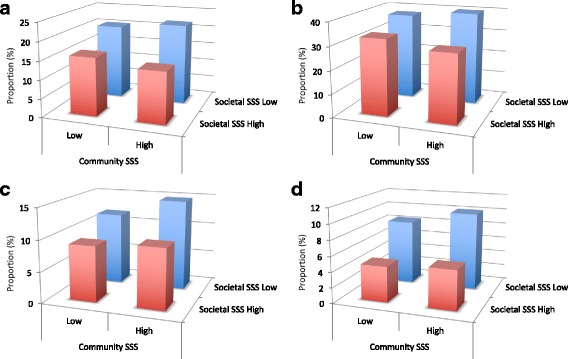
Proportions having no family doctor (**Panel a**), reporting that health is affected by lack of access to health care (**Panel b**), and experiencing all-cause (**Panel c**) and cardiac-specific (**Panel d**) readmissions to hospital within 1 year post-discharge, by societal and community subjective social status

Access to care and readmission outcomes are presented in Table 3 and Fig. 2. The low societal SSS cohort reported greater barriers to access to care compared with the high societal SSS cohort, with a higher proportion having no family physician (20.9% vs. 14.1%, *p* < 0.01), reporting transportation and the inability to leave the house as barriers to receiving routine care, and reporting that health was affected due to lack of access to care (38.4% vs. 29.7%, *p* < 0.01). The proportion of patients who were readmitted for a cardiac diagnosis within 1 year of hospital discharge was significantly higher in the low compared to the high societal SSS cohort (9.1% vs. 5.1%, *p* = 0.02) with a trend toward higher all-cause readmissions as well (13.1% vs. 9.6%, *p* = 0.10). Similarly, the odds ratios (Table 4) of not having a family doctor, reporting that health was affected by lack of health care access, and cardiac readmissions were greater than 1 for those in the low societal cohort compared with the high societal cohort (OR 1.81 [95% CI 1.31, 2.53], 1.38 [95% CI 1.02, 1.86], and 1.76 [95% CI 1.07, 2.90] respectively), adjusting for age, sex, comorbidity, and type of ACS. Though the effects were slightly attenuated when adjusting additionally for objective SES, the trends remained, with low societal SSS being associated with increased barriers to access to care and readmissions (OR 1.54 [95% CI 1.05, 2.25], 1.32 [95% CI 0.93, 1.87], and 1.76 [95% CI 0.99, 3.12] respectively for the outcomes above).

**Table 3:** Access to care and readmission outcomes by societal SSS

		Low Societal SSS N = 518, n (%)	High Societal SSS N = 572, n (%)	*P*-value
No family doctor		107 (20.9)	80 (14.1)	*< 0.01*
Difficulty accessing cardiologist		61 (15.8)	68 (15.4)	0.92
Difficulty getting routine care		101 (20.6)	104 (19.7)	0.76
Barriers in getting routine care^a^	Difficulty contacting physician	33 (27.1)	33 (29.5)	0.77
	Difficulty getting appt	67 (54.5)	69 (61.6)	0.29
	No GP	30 (25.0)	23 (20.5)	0.44
	Waited too long to get appt	41 (33.9)	40 (35.4)	0.89
	Long in office wait	37 (30.8)	31 (27.4)	0.67
	Transportation	10 (8.3)	1 (0.9)	*0.01*
	Cost	4 (3.3)	0 (0.0)	0.12
	Information	7 (5.8)	6 (5.4)	1.00
	Unable to leave house	11 (9.2)	1 (0.9)	*0.01*
	Other	8 (8.3)	3 (3.1)	0.213
Health affected by lack of access		153 (38.4)	129 (29.7)	*0.01*
Readmission within 1 year ^b^	All Cause	64 (13.0)	53 (9.6)	0.10
	Cardiac	45 (9.1)	28 (5.1)	*0.02*

^a^ Sample sizes are those indicating difficulty in getting routine care

^b^ Sample sizes at 1 year: Low societal SSS = 494; High societal SSS = 554

Abbreviations: *SSS* subjective social status; appt- appointment, *GP* general practitioner

Italics: Statistically significant difference, *p*< 0.05

**Table 4:** Logistic regression, modeling the odds of readmissions and access to care outcomes

	No family doctor	Difficulty accessing cardiologist	Difficulty accessing routine care	Health affected by lack of access	All-cause readmissions	Cardiac readmissions
Unadjusted						
High societal	Reference	Reference	Reference	Reference	Reference	Reference
Low societal	*1.61 (1.17, 2.21)*	1.03 (0.71, 1.50)	1.06 (0.78, 1.44)	*1.48 (1.11, 1.97)*	1.41 (0.96, 2.07)	*1.88 (1.15, 3.06)*
High societal/ High community	Reference	Reference	Reference	Reference	Reference	Reference
High societal/ Low community	1.16 (0.58, 2.32)	0.53 (0.20, 1.37)	1.22 (0.64, 2.30)	1.18 (0.64, 2.15)	0.92 (0.38, 2.24)	0.88 (0.26, 3.00)
Low societal/ High community	*1.74 (1.14, 2.64)*	*0.47 (0.25, 0.89)*	1.27 (0.85, 1.92)	*1.60 (1.08, 2.37)*	1.61 (0.98, 2.64)	*2.08 (1.13, 3.83)*
Low societal/ Low community	*1.58 (1.09, 2.30)*	1.33 (0.88, 2.02)	0.97 (0.67, 1.40)	*1.45 (1.03, 2.04)*	1.26 (0.79, 2.00)	1.70 (0.96, 3.02)
Adjusted for clinical and demographic factors^a^						
High societal	Reference	Reference	Reference	Reference	Reference	Reference
Low societal	*1.81 (1.31, 2.53)*	0.96 (0.64, 1.44)	0.99 (0.72, 1.35)	*1.38 (1.02, 1.86)*	1.30 (0.88, 1.93)	*1.76 (1.07, 2.90)*
High societal/ High community	Reference	Reference	Reference	Reference	Reference	Reference
High societal/ Low community	1.20 (0.60, 2.43)	0.53 (0.19, 1.45)	1.26 (0.66, 2.41)	1.11 (0.60, 2.06)	0.85 (0.34, 2.14)	0.76 (0.21, 2.75)
Low societal/ High community	*2.02 (1.31, 3.12)*	*0.46 (0.24, 0.88)*	1.20 (0.79, 1.82)	1.47 (0.98, 2.21)	1.41 (0.84, 2.34)	1.82 (0.97, 3.41)
Low societal/ Low community	*1.76 (1.20, 2.60)*	1.26 (0.81, 1.98)	0.91 (0.63, 1.33)	1.35 (0.95, 1.92)	1.19 (0.74, 1.91)	1.63 (0.91, 2.92)
Adjusted For clinical, demographic, and socioeconomic factors^b^						
High societal	Reference	Reference	Reference	Reference	Reference	Reference
Low societal	*1.54 (1.05, 2.25)*	0.79 (0.50, 1.26)	1.07 (0.75, 1.53)	1.32 (0.93, 1.87)	1.28 (0.81, 2.02)	1.76 (0.99, 3.12)
High societal/ High community	Reference	Reference	Reference	Reference	Reference	Reference
High societal/ Low community	1.00 (0.45, 2.24)	0.62 (0.22, 1.71)	1.46 (0.72, 2.96)	1.00 (0.50, 1.98)	0.84 (0.30, 2.33)	0.53 (0.11, 2.61)
Low societal/ High community	1.63 (1.00, 2.66)	*0.38 (0.18, 0.80)*	1.33 (0.84, 2.11)	1.36 (0.86, 2.16)	1.32 (0.74, 2.36)	1.57 (0.76, 3.25)
Low societal/ Low community	1.48 (0.95, 2.30)	1.04 (0.62, 1.74)	0.99 (0.65, 1.51)	1.29 (0.87, 1.93)	1.21 (0.71, 2.07)	1.72 (0.90, 3.29)

^*a*^ Adjusted for age, sex, comorbidity count, and type of acute coronary syndrome

^b^ Adjusted for age, sex, comorbidity count, type of acute coronary syndrome, household income, employment status

Italics: Statistically significant, with the 95% confidence interval not crossing 1

### Concordant versus discordant community rankings within the low societal SSS cohort

Within the low societal SSS cohort, those with concordantly low community SSS rankings had similar baseline demographics and clinical characteristics compared to those with discordantly high community rankings (Additional file 1: Tables S1 and S2). However, there were significant differences in access to care outcomes (Table 5 and Fig. 2), with a greater proportion of those with concordantly low community rankings reporting difficulty accessing a cardiologist compared to those with discordantly high community rankings (20.6% vs. 8.4%, *p* < 0.01). Of those reporting difficulty accessing routine care, a higher proportion of those with concordantly low community rankings reported that this difficulty was due to transportation, cost, and information barriers, as well as inability to leave the house due to the medical condition. These differences were not statistically significant, likely due to the low numbers reporting difficulty accessing routine care. There were no statistically significant differences in the readmission rates between those with low versus high community rankings.

**Table 5:** Access to care and readmission outcomes by societal and community SSS concordance

	Low Societal SSS			High Societal SSS		
	Concordantly Low Community SSS *N* = 314, n (%)	Discordantly High Community SSS *N* = 204, n (%)	*P*-value	Concordantly High Community SSS *N* = 502, n (%)	Discordantly Low Community SSS *N* = 70, n (%)	*P*-value
No family doctor	63 (20.3)	44 (21.8)	0.74	69 (13.8)	11 (15.7)	0.71
Difficulty accessing cardiologist	48 (20.6)	13 (8.4)	*< 0.01*	63 (16.3)	5 (9.3)	0.23
Difficulty getting routine care	57 (18.9)	44 (23.4)	0.25	90 (19.4)	14 (22.6)	0.61
Barriers in getting routine care^a^	20 (29.4)	13 (24.1)	0.55	29 (29.9)	4 (26.7)	1.00
	38 (55.9)	29 (52.7)	0.86	60 (61.9)	9 (60.0)	1.00
	13 (19.7)	17 (31.5)	0.15	19 (19.6)	4 (26.7)	0.51
	20 (29.9)	21 (38.9)	0.34	32 (32.7)	8 (53.3)	0.15
	20 (30.3)	17 (31.5)	1.00	25 (25.5)	6 (40.0)	0.35
	8 (11.9)	2 (3.7)	0.18	1 (1.0)	0 (0.0)	1.00
	3 (4.6)	1 (1.9)	0.63	0 (0.0)	0 (0.0)	1.00
	6 (9.1)	1 (1.9)	0.13	5 (5.2)	1 (6.7)	0.59
	9 (13.6)	2 (3.7)	0.11	1 (1.0)	0 (0.0)	1.00
	4 (7.7)	4 (8.9)	1.00	3 (3.6)	0 (0.0)	1.00
Health affected by lack of access	92 (37.6)	61 (39.9)	0.67	111 (29.3)	18 (32.7)	0.64
Readmission within 1 year ^b^	35 (11.8)	29 (14.7)	0.41	47 (9.7)	6 (9.0)	1.00
	25 (8.4)	20 (10.1)	0.53	25 (5.1)	3 (4.6)	1.00

Abbreviations: *SSS* subjective social status, appt- appointment, *GP* general practitioner

^a^ Sample sizes are those indicating difficulty in getting routine care

^b^ Sample sizes at 1 year: Low societal/Low community = 296; Low societal/High community = 198; High societal/High community = 487; High societal/Low community = 67

Italics: Statistically significant difference, *p*< 0.05

The odds of experiencing any of the barriers to health care access and readmission outcomes were not significantly different when comparing patients with concordantly low community rankings to those with discordantly high rankings within the low societal SSS cohort (Table 4). If having high community SSS were to mitigate the access to care barriers and readmissions, one would expect the odds ratios for these outcomes to be greater for the low societal with concordantly low community rankings group compared to the low societal with discordantly high community rankings group (Table 4). This was not found to be the case, with the 95% confidence intervals for the odds ratios for these groups overlapping, and with no clear pattern demonstrating that one group had higher odds of worse outcomes.

### Concordant versus discordant community rankings within the high societal SSS cohort

Within the high societal SSS cohort, patients with concordantly high community SSS rankings were similar to those patients with discordantly low community SSS rankings in terms of baseline demographics and clinical characteristics (Additional file 1: Tables S1 and S2). There were no statistically significant differences in access to care and readmission rates (Table 5 and Fig. 2) or in the odds ratios of these outcomes between the concordant versus discordant community ranking groups (Table 4).

## Discussion

In a cohort of hospitalized patients with acute coronary syndrome, low societal subjective social status is associated with increased barriers to access to care, increased cardiac readmissions, and a trend toward increased all-cause readmissions. Subjective social status then, appears to be an important measure that not only has implications for the risk of developing cardiovascular disease, but also has prognostic implications in those with established disease. This leads to fundamental questions about what SSS actually measures and how it links to cardiovascular health.

There are three main hypotheses in the literature regarding the general SSS construct and its relationship to health. The first is that SSS is a general self-rank of social status based on the average of one’s socioeconomic contributions [9, 27]. An example used in the SSS literature is that a high school graduate from an inner-city school may not have the same life chances as a student with extensive family resources graduating from an elite prep school, yet objective SES measures would consider them to have the same social status [9]. SSS may therefore be a more comprehensive measure of overall SES taking into account past and future resource trajectories. A second related hypothesis is that SSS captures not only objective socioeconomic measures but also non-objective measures of self-worth and social position. In a qualitative study, a majority of participants indicated that non-objective measures such as values and altruism contributed to their perceived worth and social position in the community [9].

A third hypothesis is that SSS captures the psychosocial processes that mediate the association between SES and health outcomes, rather than being just another measure of SES. It posits that SES affects health not just in the resources that can be accessed, but that SES serves as “reference points for social comparison” [28]. Perceptions of inferiority in a social hierarchy may have psychosocial consequences by acting as a source of chronic stress, and by affecting optimism/pessimism, sense of mastery, social supports, and ability to cope with life stressors [29, 30]. Low societal and community SSS have both been shown to be associated with reduced endothelial function and impaired vasodilation, increased cortisol production, and reduced beta adrenergic receptor responsiveness [31–33]; these changes are consistent with activation of neuroendocrine stress pathways. Our study findings lend support to this third hypothesis, by showing that non-socioeconomic attributes such as social support differ between the high and low SSS cohorts; additionally, when objective SES measures are added to regression models, there is attenuation of the associations between SSS and access to health and readmission outcomes, which would be expected if SES and SSS are on the same causal pathway. Despite this attenuation, a consistent trend remains, which suggests that SSS has independent associations on health and health care access over and above what is captured by traditional SES measures. This would also support the first two aforementioned hypotheses. Our study findings therefore lend support to all three hypotheses of the mechanisms that link SSS to cardiovascular health.

The relationship between rankings on the societal versus community SSS ladders, and the association of this relationship with health outcomes has not been previously established. We have developed a novel method of examining societal and community SSS rankings simultaneously in a way that captures their relative positions; in doing so, we have found that the two ladders appear to be distinct constructs. First, objective SES measures do not seem to weigh heavily into community ladder rankings, though they do for societal ladder rankings. When the study sample was divided into low versus high societal SSS, there was no further difference in income, education, or employment for those with low versus high community rankings. Second, our findings suggest that the societal scale may take into account more than objective SES measures to reflect psychosocial contributions as well, with those in the high societal SSS cohort reporting greater social support compared to the low societal SSS cohort. Also, differences in rankings of the community scale cannot be ascribed purely to differences in social support as captured by the ESSI, as there were no further differences in ESSI scores between high and low community SSS patients within either the low or the high societal SSS cohorts.

The two SSS ladders also seem to have unique associations with access to care and hospital readmissions in patients with ACS. Though results were variable based on the outcome studied, high community rankings showed a trend in possibly modifying some of the access to care barriers in those with low societal SSS, though there did not seem to be any modifying effects for those with high societal SSS. Community SSS also did not modify the association between societal SSS and readmissions, contrary to our hypothesis. There may be numerous reasons for this. Higher social trust, reciprocity, social capital, and social connectedness are associated with improved health outcomes [34], but these concepts may not be accurately represented by the MacArthur community ladder; residents of neighbourhoods with high social cohesion may consider themselves more or less as equals with their neighbours and therefore not rank themselves highly. Secondly, there is no consensus as to what community SSS rankings actually capture. Based on preliminary data, it seems that social capital is one characteristic that may determine community SSS rankings [9], but social capital in itself is a vague and difficult-to-measure concept. A systematic review on the association between social capital and health care access has shown inconsistent effects, with numerous definitions and methods of operationalizing social capital [35]; no single indicator of social capital is consistently associated with improved health care access across studies. To add to this confusion, community SSS likely encompasses not only social capital, but also social relationships, self-esteem, self-worth, and psychosocial factors such as optimism, stress, and anxiety [10, 22, 36]. The lack of association in our study between community SSS and health access and readmissions is not surprising then, given the heterogeneous concepts captured in this ladder. The ladder is known to have lower test-retest stability compared to the societal ladder [26], and rankings are also less predictable on this ladder compared to the societal ladder [37]. Though the community SSS ladder captures some psychosocial aspect of social status, these aspects are likely heterogeneous and the use of a summary measure likely results in the variable and weakened associations found with access to care and readmission outcomes.

The fundamental limitation to our study is inherent to the topic of interest, with SSS being an indistinct and mysterious concept. Though the theoretical basis of SSS is well-defined, how individuals choose to rank themselves on a social hierarchy, how this differs between the two SSS ladders, and what psychosocial characteristics these rankings represent remain uncertain. This ambiguity limits our analysis and interpretation of our findings, but also serves to highlight the difficulty in undertaking research around the complex construct of subjective social status. Other specific limitations include being underpowered to detect the type of barriers to access of care despite appropriate power to detect clinical outcomes of readmissions, possible misclassification bias as readmissions were identified through self-report, and our inclusion criterion of individuals needing to be fluent in English or French. This inclusion criterion may have excluded those with least access to care and lowest SSS, resulting in an underestimation of the association between low SSS and access to care and readmission outcomes in patients with ACS in our study. Our relatively young and predominantly Canadian cohort may limit generalizability of our findings. However, low objective socioeconomic status has repeatedly been demonstrated to be associated with cardiovascular disease across different populations [1, 38]. Given the overlap between subjective social status and socioeconomic status, and that both are measures of social status, our findings likely also apply more broadly. Lastly, hospital readmissions may be attributable to a complex interplay of patient-level, environmental, and organizational factors [39]. Though we adjusted for important patient-level confounders, there are likely environmental and organizational factors that may contribute to hospital readmissions that we were unable to take into account.

Subjective social status appears to be a social determinant of health, with previous studies showing associations between low SSS and mental health, self-rated health, and cardiovascular disease. We show that that low SSS is associated with barriers to access to care and higher cardiac readmissions in patients with established coronary artery disease. Furthermore, our findings suggest that subjective social status is not a homogenous construct, with societal and community SSS representing distinct concepts. This distinction has significant implications for the development of interventions. For example, programs that address the former include investment in human capital such as job training and job creation; interventions that address the latter include increasing social capital through improving community supports and community engagement. Future research will need to focus on clarifying the similarities and differences between the societal and community SSS constructs and their associations with health, as this is imperative in informing the prioritization and development for public health interventions to improve cardiovascular health of the population.

## Additional files

Additional file 1: **Table S1.** Baseline demographics by societal and community SSS concordance; **Table S2.** Baseline clinical characteristics by societal and community SSS concordance. (DOCX 24 kb)

Additional file 2: Appendix 1. Co-investigators for GENESIS-PRAXY. (DOCX 15 kb)

Additional file 3: Appendix 2. GENESIS-PRAXY Participating Centres. (DOCX 15 kb)

## Abbreviations

ACS: Acute coronary syndrome
BMI: Body mass index
CI: Confidence interval
CK-MB: Creatine kinase – MB isoenzyme
CPK: Creatine phosphokinase
ECG: Electrocardiogram
ESSI: ENRICHD Social Support Instrument
GENESIS-PRAXY: Gender and sex determinants of cardiovascular disease: from bench to beyond premature acute coronary syndrome
GP: General practitioner
IQR: Interquartile range
LV function: Left ventricular function
MI: Myocardial infarction
OR: Odds ratio
PCI: Percutaneous coronary intervention
SD: Standard deviation
SES: Socioeconomic status
SSS: Subjective social status

## References

[1] Kaplan GA, Keil JE (1993). Socioeconomic factors and cardiovascular disease: a review of the literature. Circulation.

[2] Alter DA, Franklin B, Ko DT, Austin PC, Lee DS, Oh PI, Stukel TA, Tu JV (2014). Socioeconomic status, functional recovery, and long-term mortality among patients surviving acute myocardial infarction. PLoS One.

[3] Bernheim SM, Spertus JA, Reid KJ, Bradley EH, Desai RA, Peterson ED, Rathore SS, Normand SL, Jones PG, Rahimi A (2007). Socioeconomic disparities in outcomes after acute myocardial infarction. Am Heart J.

[4] Lindenauer PK, Lagu T, Rothberg MB, Avrunin J, Pekow PS, Wang Y, Krumholz HM (2013). Income inequality and 30 day outcomes after acute myocardial infarction, heart failure, and pneumonia: retrospective cohort study. BMJ (Clin Res Ed).

[5] Shah SJ, Krumholz HM, Reid KJ, Rathore SS, Mandawat A, Spertus JA, Ross JS (2012). Financial stress and outcomes after acute myocardial infarction. PLoS One.

[6] Edmondson D, Green P, Ye S, Halazun HJ, Davidson KW (2014). Psychological stress and 30-day all-cause hospital readmission in acute coronary syndrome patients: an observational cohort study. PLoS One.

[7] Marmot MG, Smith GD, Stansfeld S, Patel C, North F, Head J, White I, Brunner E, Feeney A (1991). Health inequalities among British civil servants: the Whitehall II study. Lancet.

[8] Jackman MR, Jackman RW (1973). An interpretation of the relation between objective and subjective social status. Am Sociol Rev.

[9] The MacArthur Scale of Subjective Social Status [http://www.macses.ucsf.edu/research/psychosocial/subjective.php].

[10] Ghaed SG, Gallo LC (2007). Subjective social status, objective socioeconomic status, and cardiovascular risk in women. Health Psychol.

[11] Adler N, Singh-Manoux A, Schwartz J, Stewart J, Matthews K, Marmot MG (2008). Social status and health: a comparison of British civil servants in Whitehall-II with European- and African-Americans in CARDIA. Soc Sci Med.

[12] Demakakos P, Marmot M, Steptoe A (2012). Socioeconomic position and the incidence of type 2 diabetes: the ELSA study. Eur J Epidemiol.

[13] Demakakos P, Nazroo J, Breeze E, Marmot M (2008). Socioeconomic status and health: the role of subjective social status. Soc Sci Med.

[14] Frerichs L, Huang TT, Chen DR (2014). Associations of subjective social status with physical activity and body mass index across four Asian countries. J Obes.

[15] Manuck SB, Phillips JE, Gianaros PJ, Flory JD, Muldoon MF (2010). Subjective socioeconomic status and presence of the metabolic syndrome in midlife community volunteers. Psychosom Med.

[16] Singh-Manoux A, Adler NE, Marmot MG (2003). Subjective social status: its determinants and its association with measures of ill-health in the Whitehall II study. Soc Sci Med.

[17] Woo J, Lynn H, Leung J, Wong SY (2008). Self-perceived social status and health in older Hong Kong Chinese women compared with men. Women Health.

[18] Tang KL, Rashid R, Godley J, Ghali WA (2016). Association between subjective social status and cardiovascular disease and cardiovascular risk factors: a systematic review and meta-analysis. BMJ Open.

[19] Shanmugasegaram S, Oh P, Reid RD, McCumber T, Grace SL (2013). Cardiac rehabilitation barriers by rurality and socioeconomic status: a cross-sectional study. Int J Equity Health.

[20] Tsui CK, Shanmugasegaram S, Jamnik V, Wu G, Grace SL (2012). Variation in patient perceptions of healthcare provider endorsement of cardiac rehabilitation. J Cardiopulm Rehab Prev.

[21] Das S, O'Keefe JH (2006). Behavioral cardiology: recognizing and addressing the profound impact of psychosocial stress on cardiovascular health. Curr Atheroscler Rep.

[22] Gage-Bouchard EA, Devine KA (2014). Examining parents’ assessments of objective and subjective social status in families of children with cancer. PLoS One.

[23] Uphoff EP, Pickett KE, Cabieses B, Small N, Wright J (2013). A systematic review of the relationships between social capital and socioeconomic inequalities in health: a contribution to understanding the psychosocial pathway of health inequalities. Int J Equity Health.

[24] GRACE Variable Definitions—Version of 2006 [https://www.outcomes-umassmed.org/grace/publicfiles/Standard_Definitions.pdf].

[25] Pilote L, Karp I (2012). GENESIS-PRAXY (GENdEr and sex determInantS of cardiovascular disease: from bench to beyond-premature acute coronary SYndrome). Am Heart J.

[26] Giatti L, do Valle Camelo L, de Castro Rodrigues JF, Barreto SM (2012). Reliability of the MacArthur scale of subjective social status-Brazilian longitudinal study of adult health (ELSA-Brasil). BMC Public Health.

[27] Nobles J, Weintraub MR, Adler NE (2013). Subjective socioeconomic status and health: relationships reconsidered. Soc Sci Med (1982).

[28] Schnittker J, McLeod JD. The social psychology of health disparities. Ann Rev Sociol. 2005:75–103.

[29] Taylor SE, Seeman TE (1999). Psychosocial resources and the SES-health relationship. Ann N Y Acad Sci.

[30] Adler NE, Epel ES, Castellazzo G, Ickovics JR (2000). Relationship of subjective and objective social status with psychological and physiological functioning: preliminary data in healthy white women. Health Psychol.

[31] Cooper DC, Milic MS, Mills PJ, Bardwell WA, Ziegler MG, Dimsdale JE (2010). Endothelial function: the impact of objective and subjective socioeconomic status on flow-mediated dilation. Ann Behav Med.

[32] Euteneuer F, Mills PJ, Rief W, Ziegler MG, Dimsdale JE (2012). Subjective social status predicts in vivo responsiveness of beta-adrenergic receptors. Health Psychol.

[33] Gruenewald TL, Kemeny ME, Aziz N (2006). Subjective social status moderates cortisol responses to social threat. Brain Behav Immunity.

[34] Putnam RD (2000). Bowling Alone.

[35] Derose KP, Varda DM (2009). Social capital and health care access: a systematic review. Med Care Res Rev: MCRR.

[36] Holt-Lunstad J, Smith TB, Layton JB (2010). Social relationships and mortality risk: a meta-analytic review. PLoS Med.

[37] Gage E (2011). Abstract B20: examining measures of socioeconomic status for cancer disparities research. Cancer Epidemiol Biomark Prev.

[38] Clark AM, DesMeules M, Luo W, Duncan AS, Wielgosz A (2009). Socioeconomic status and cardiovascular disease: risks and implications for care. Nat Rev Cardiol.

[39] Vest JR, Gamm LD, Oxford BA, Gonzalez MI, Slawson KM (2010). Determinants of preventable readmissions in the United States: a systematic review. Implementation Sci: IS.

