# Supplementary material for: An exploration of the subjective social status construct in patients with acute coronary syndrome
Source: BMC Cardiovasc Disord. 2018 Feb 6;18:22. doi: 10.1186/s12872-018-0759-7 (PMC5801903; doi:10.1186/s12872-018-0759-7)
Supplement: Supplementary file 1 — Table S1. Baseline demographics by societal and community SSS concordance; Table S2. Baseline clinical characteristics by societal and community SSS concordance. (DOCX 24 kb) [file 12872_2018_759_MOESM1_ESM.docx]

**Table S1 Baseline demographics by societal and community SSS concordance**

|  |  | Low Societal SSS | | | High Societal SSS | | |
| --- | --- | --- | --- | --- | --- | --- | --- |
|  |  | Concordantly Low Community SSS  N= 314, n(%) | Discordantly High Community SSS  N= 204, n(%) | P value | Concordantly High Community SSS  N= 502, n(%) | Discordantly Low Community SSS  N= 70, n(%) | P-value |
| Age (years) | Mean (SD) | 47.9 (6.1) | 48.3 (5.6) | 0.45 | 48.3 (5.8) | 48.1 (5.6) | 0.79 |
| Female |  | 119 (37.9) | 73 (35.8) | 0.64 | 135 (26.9) | 19 (27.1) | 1.00 |
| Ethnicity | Caucasian | 263 (85.1) | 173 (86.9) | 0.12 | 431 (88.1) | 65 (94.2) | 0.09 |
|  | Aboriginal | 9 (2.9) | 9 (4.5) |  | 10 (2.0) | 3 (4.4) |  |
|  | Chinese | 35 (11.3) | 13 (6.5) |  | 38 (7.8) | 1(1.5) |  |
|  | Other | 2 (0.7) | 4 (2.0) |  | 10 (2.0) | 0 (0.0) |  |
| First Language | English | 164 (55.0) | 124 (62.3) | 0.26 | 282 (58.5)  141 (29.3)  59 (12.2) | 53 (76.8)  12 (17.4)  4 (5.8) | **0.01** |
|  | French | 79 (26.5) | 46 (23.1) |  |  |  |  |
|  | Other | 55 (18.5) | 29 (14.6) |  |  |  |  |
| Current Smoker |  | 150 (47.8) | 101 (49.5) | 0.72 | 166 (33.1) | 21 (30.0) | 0.68 |
| Low Household Income | <$50 000 | 133 (50.4) | 88 (51.5) | 0.85 | 90 (20.9) | 9 (15.5) | 0.39 |
| Education | No degree, certificate, diploma | 51 (16.5) | 28 (14.0) | 0.86 | 43 (8.6) | 8 (11.6) | 0.16 |
|  | High School Diploma | 86 (27.8) | 57 (28.5) |  | 114 (22.9) | 14 (20.3) |  |
|  | Some Post-Secondary | 64 (20.7) | 41 (20.5) |  | 94 (18.9) | 8 (11.6) |  |
|  | Completed Post-Secondary | 72 (23.3) | 45 (22.5) |  | 183 (36.8) | 34 (49.3) |  |
|  | Trades Certificate | 36 (11.7) | 29 (14.5) |  | 64 (12.9) | 5 (7.3) |  |
| Employment Status | Currently working | 218 (69.4) | 153 (75.0) | 0.20 | 440 (87.7) | 60 (85.7) | 0.70 |
|  | Student | 5 (1.6) | 4 (2.0) | 0.74 | 8 (1.6) | 0 (0.0) | 0.61 |
|  | Homemaker | 19 (6.1) | 12 (5.9) | 1.00 | 16 (3.2) | 2 (2.9) | 1.00 |
|  | Unemployed | 32 (10.2) | 14 (6.9) | 0.21 | 13 (2.6) | 4 (5.7) | 0.14 |
|  | Leave of Absence | 45 (14.3) | 21 (10.3) | 0.23 | 31 (6.2) | 1 (1.4) | 0.16 |
|  | Disabled | 5 (1.6) | 2 (1.0) | 0.71 | 2 (0.4) | 1 (1.4) | 0.33 |
|  | Retired | 5 (1.6) | 3 (1.5) | 1.00 | 9 (1.8) | 2 (2.9) | 0.63 |
| Live alone |  | 69 (22.0) | 37 (18.1) | 0.32 | 61 (12.2) | 5 (7.1) | 0.32 |
| Social Support ESSI Sum Score | Mean (SD) | 27.1 (7.4) | 26.8 (7.2) | 0.58 | 29.8 (5.9) | 29.3 (6.0) | 0.49 |

Abbreviations: SSS- subjective social status; SD- standard deviation; ESSI- ENRICHD Social Support Inventory

**Table S2 Baseline clinical characteristics by societal and community SSS concordance**

|  |  | Low Societal SSS | | | High Societal | | |
| --- | --- | --- | --- | --- | --- | --- | --- |
|  |  | Concordantly Low Community SSS  N= 314, n(%) | Discordantly High Community SSS  N= 204, n(%) | P value | Concordantly High Community SSS  N= 502, n(%) | Discordantly Low Community SSS  N= 70, n(%) | P value |
| BMI (kg/m^2^) | Mean (SD) | 29.7 (7.2) | 30.2 (7.1) | 0.41 | 29.1 (5.3) | 30.1 (7.7) | 0.18 |
| Type of MI on admission | STEMI | 167 (53.2) | 124 (60.8) | 0.10 | 299 (59.6) | 45 (64.3) | 0.52 |
|  | NSTEMI | 123 (39.2) | 66 (32.4) | 0.14 | 158 (31.5) | 15 (21.4) | 0.10 |
|  | Unstable Angina | 18 (5.7) | 10 (4.9) | 0.84 | 41 (8.2) | 9 (12.9) | 0.18 |
| Reperfusion |  | 238 (77.0) | 157 (78.5) | 0.75 | 390 (78.5) | 54 (79.4) | 1.00 |
| Method of Reperfusion | Primary PCI | 102 (42.3) | 80 (47.6) | 0.31 | 193 (47.3) | 32 (55.2) | 0.27 |
|  | Non Primary PCI | 129 (53.5) | 76 (45.5) | 0.13 | 192 (47.1) | 24 (41.4) | 0.48 |
|  | Thrombolytics | 41 (17.0) | 28 (16.8) | 1.00 | 64 (15.7) | 7 (12.1) | 0.56 |
| Peak Troponin T, Mean (SD) |  | 6.5 (20.7) | 11.2 (35.6) | 0.28 | 6.5 (19.7) | 3.9 (4.1) | 0.51 |
| Comorbidities | Angina | 98 (31.2) | 76 (37.3) | 0.18 | 147 (29.3) | 29 (41.4) | 0.05 |
|  | Cancer | 3 (1.0) | 2 (1.0) | 1.00 | 16 (3.2) | 1 (1.4) | 0.71 |
|  | Diabetes | 56 (17.8) | 43 (21.1) | 0.36 | 65 (13.0) | 6 (8.6) | 0.44 |
|  | Congestive heart failure | 7 (2.2) | 3 (1.5) | 0.78 | 10 (2.0) | 0 (0.0) | 0.62 |
|  | Hypertension | 120 (38.2) | 87 (42.7) | 0.36 | 186 (37.1) | 22 (31.4) | 0.43 |
|  | Hyperthyroid | 3 (1.0) | 4 (2.0) | 0.44 | 9 (1.8) | 1 (1.4) | **0.03** |
|  | Dyslipidemia | 129 (41.1) | 95 (46.6) | 0.24 | 202 (40.2) | 26 (37.1) | 0.70 |
|  | Peripheral Artery Disease | 9 (2.9) | 4 (2.0) | 0.58 | 4 (0.8) | 1 (1.4) | 0.48 |
|  | Depression | 36 (12.1) | 26 (13.8) | 0.58 | 41 (8.6) | 3 (4.6) | 0.34 |
|  | Renal Disease | 5 (1.6) | 2 (1.0) | 0.71 | 7 (1.4) | 3 (4.3) | 0.11 |
|  | Previous MI | 47 (15.0) | 28 (13.7) | 0.80 | 54 (10.8) | 6 (8.6) | 0.68 |
| LV Function (%) | Mean (SD) | 51.0 (11.0) | 51.2 (12.3) | 0.87 | 51.4 (9.9) | 48.3 (9.1) | 0.05 |
| Complications in hospital | Atrial fibrillation | 9 (2.9) | 3 (1.5) | 0.38 | 8 (1.6) | 0 (0.0) | 0.61 |
|  | Angina | 16 (5.1) | 9 (4.4) | 0.84 | 23 (4.6) | 4 (5.7) | 0.56 |
|  | Bradycardia | 7 (2.2) | 5 (2.5) | 1.00 | 9 (1.8) | 1 (1.4) | 1.00 |
|  | Cardiogenic Shock | 1 (0.3) | 2 (1.0) | 0.57 | 5 (1.0) | 0 (0.0) | 1.00 |
|  | Hypotension | 6 (1.9) | 8 (3.9) | 0.18 | 15 (3.0) | 2 (2.9) | 1.00 |
|  | Ventricular tachycardia | 14 (4.5) | 11 (5.4) | 0.68 | 16 (3.2) | 1 (1.4) | 0.71 |
|  | Ventricular fibrillation | 11 (3.5) | 4 (2.0) | 0.42 | 18 (3.6) | 2 (2.9) | 1.00 |
|  | Congestive heart failure | 5(1.6) | 1 (0.5) | 0.41 | 9 (1.8) | 3 (4.3) | 0.17 |
|  | Pericarditis | 1 (0.3) | 3 (1.5) | 0.31 | 3 (0.6) | 1 (1.4) | 0.41 |
|  | Reinfarction | 2 (0.6) | 2 (1.0) | 0.65 | 1 (0.2) | 1 (1.4) | 0.23 |

Abbreviations: SSS- subjective social status; SD- standard deviation; BMI- body mass index; PCI- percutaneous coronary intervention; MI-myocardial infarction; LV- left ventricular dysfunction
