# Supplementary material for: An exploration of the subjective social status construct in patients with acute coronary syndrome
Source: BMC Cardiovasc Disord. 2018 Feb 6;18:22. doi: 10.1186/s12872-018-0759-7 (PMC5801903; doi:10.1186/s12872-018-0759-7)
Supplement: Supplementary file 2 — Appendix 1. Co-investigators for GENESIS-PRAXY. (DOCX 15 kb) [file 12872_2018_759_MOESM2_ESM.docx]

**Additional file 2: Appendix 1** Co-Investigators for GENESIS-PRAXY

| **Co-Principal Investigators** |
| --- |
| Louise Pilote (MD, MPH, PhD), Divisions of General Internal Medicine and Clinical Epidemiology, McGill University Health Centre, Montréal, QC, Canada |
| Igor Karp (MD, MPH, PhD), Department of Epidemiology and Biostatistics, Schulich School of Medicine and Dentistry, University of Western ON, Canada and University of Montréal Hospital Research Centre (CRCHUM) and Department of Social and Preventive Medicine, University of Montréal, Montréal, QC, Canada |
| **Co-Investigators** |
| Simon L. Bacon (PhD), Concordia University and Research Centre, Hôpital du Sacré-Coeur de Montréal, Montréal, QC, Canada |
| Jafna L. Cox (BA, MD, FRCPC, FACC), Department of Medicine and of Community Health and Epidemiology, Dalhousie University, Halifax, NS, Canada |
| Kaberi Dasgupta (MD, MSc, FRCPC), Research Institute of the McGill University Health Centre, Montréal, QC, Canada |
| Stella S. Daskalopoulou (MD, MSc, PhD), Research Institute of the McGill University Health Centre, Montréal, QC, Canada |
| Mark J. Eisenberg (MD, MPH) Jewish General Hospital, McGill University, Montréal, QC, Canada |
| James C. Engert (PhD), Research Institute of the McGill University Health Centre, Montréal, QC, Canada |
| William A. Ghali (MD, MPH, FRCPC), University of Calgary, Calgary, AB, Canada |
| Karin H. Humphries (MBA DSc), University of British Columbia, Vancouver, BC, Canada |
| Nadia A. Khan (MD, MSc), University of British Columbia, Vancouver, BC, Canada |
| Kim L. Lavoie (PhD), University of Quebec at Montréal (UQAM) and Research Centre, Hôpital du Sacré-Coeur de Montréal, Montréal, QC, Canada |
| Colleen M. Norris (RN, PhD), University of Alberta, Edmonton, AB, Canada |
| Doreen Rabi (MD, FRCPC, MS), University of Calgary, Calgary, AB, Canada |
| Derek So (MD, FRCPC, FACC), University of Ottawa Heart Institute, Ottawa, ON, Canada |
| Ken D. Stark (PhD), Department of Kinesiology, University of Waterloo, Waterloo, ON, Canada |
| Vicky Tagalakis (MD, FRCPC, MSc), McGill University, Divisions of Internal Medicine and Centre for Clinical Epidemiology and Community Studies, Jewish General Hospital, Montréal, QC, Canada |
| George Thanassoulis (MD, FRCPC) Research Institute of the McGill University Health Centre, Montréal, QC, Canada |
