# Supplementary material for: An exploration of the subjective social status construct in patients with acute coronary syndrome
Source: BMC Cardiovasc Disord. 2018 Feb 6;18:22. doi: 10.1186/s12872-018-0759-7 (PMC5801903; doi:10.1186/s12872-018-0759-7)
Supplement: Supplementary file 3 — Appendix 2. GENESIS-PRAXY Participating Centres. (DOCX 15 kb) [file 12872_2018_759_MOESM3_ESM.docx]

**Additional file 3: Appendix 2** GENESIS-PRAXY Participating Centres

| **Site** | **Site PI** |
| --- | --- |
| St Paul's Hospital, Vancouver, British Columbia, Canada | Krishan Ramanthan |
| Surrey Memorial Hospital, Surrey, British Columbia, Canada | Jan Kornder |
| Libin Cardiovascular Institute of Alberta, University of Calgary, Calgary, Alberta, Canada | Todd Anderson  /Doreen Rabi |
| University of Alberta and the Mazankowski Alberta Heart Institute, Edmonton, Alberta, Canada | Colleen Norris  /Michelle Graham |
| University of Ottawa Heart Institute, Ottawa, Ontario, Canada | Derek So |
| McMaster University/Hamilton Health Sciences (General Site), Hamilton, Ontario, Canada | Madhu Natarajan |
| McMaster University/Hamilton Health Sciences (Juravinski Site), Hamilton, Ontario, Canada | Mike Rokoss |
| Ottawa Hospital, Ottawa, Ontario, Canada | Michele Turek |
| St Michael's Hospital, Toronto, Ontario, Canada | Asim Cheema |
| London Health Sciences Centre, London, Ontario, Canada | Shahar Lavi |
| The Scarborough Hospital, General Division, Scarborough, Ontario, Canada | Sherryn Roth |
| Hôpital Général de Montréal, Montréal, Québec, Canada | Thao Huynh |
| Hôpital Royal Victoria, Montréal, Québec, Canada | Viviane Nguyen |
| Hôpital Général Juif-Sir Mortimer B. Davis, Montréal, Québec, Canada | Mark Eisenberg |
| Institut universitaire de cardiologie et de pneumologie de Québec (Hôpital Laval), Québec, Québec, Canada | Julie Méthot |
| Hôpital du Sacré-Coeur de Montréal, Montréal, Québec, Canada | Michel Doucet |
| Cité de la Santé de Laval, Laval, Québec, Canada | Martine Montigny |
| Hôtel Dieu du Centre Hospitalier de l'Université de Montréal, Montréal, Québec, Canada | Samer Mansour |
| Centre de santé et de services sociaux de la région de Thetford, Thetford Mines, Québec, Canada | Claude Lauzon |
| CSSS Chicoutimi, Chicoutimi, Québec, Canada | Tomas Cieza |
| Centre Hospitalier Universitaire de Sherbrooke, Sherbrooke, Québec, Canada | Michel Nguyen |
| CSSS Alphonse Desjardins (CHAU - Hôtel-Dieu de Lévis), Lévis, Québec, Canada | François Grondin |
| Queen Elizabeth II Health Science Centre, Halifax, Nova Scotia, Canada | Jafna Cox |
| The New Brunswick Heart Centre Research Initiative and The New Brunswick Heart Centre, New Brunswick, Canada | Peter Fong |
| Basset Healthcare, Cooperstown, New-York, USA | Dhananjai Menzies |
| Inselspital, University of Bern, Switzerland and Lausanne University Hospital, Lausanne, Switzerland | Nicolas Rodondi |
